# Pulmonary torsion after resuscitative thoracotomy: a case report

**DOI:** 10.1093/jscr/rjab313

**Published:** 2021-07-19

**Authors:** Daisuke Hara, Tomoka Hamahiro, Ryo Maeda, Takanori Ayabe, Masaki Tomita

**Affiliations:** Department of Thoracic and Breast Surgery, Faculty of Medicine, University of Miyazaki, Miyazaki, 889-1692, Japan; Department of Thoracic and Breast Surgery, Faculty of Medicine, University of Miyazaki, Miyazaki, 889-1692, Japan; Department of Thoracic and Breast Surgery, Faculty of Medicine, University of Miyazaki, Miyazaki, 889-1692, Japan; Department of Thoracic and Breast Surgery, Faculty of Medicine, University of Miyazaki, Miyazaki, 889-1692, Japan; Department of Thoracic and Breast Surgery, Faculty of Medicine, University of Miyazaki, Miyazaki, 889-1692, Japan

## Abstract

We report a rare case of pulmonary torsion after nonpulmonary thoracotomy. A 38-year-old woman with schizophrenia committed suicide by a self-infliction of sharp force into the chest and abdomen. During emergent abdominal damage control surgery, a left-sided resuscitative thoracostomy was also performed due to hemorrhagic shock. Although abnormal shadow was detected on postoperative chest roentgenogram and computed tomography, the diagnosis of pulmonary torsion was delayed. Seven days after initial surgery, pulmonary torsion was diagnosed and managed by left upper lobectomy. To our knowledge, this is the first report of pulmonary torsion after resuscitative thoracotomy.

## INTRODUCTION

Pulmonary torsion is a rare condition, most commonly seen as a complication after lobectomy and life-threatening disease that requires a timely diagnosis [1–3]. A case with pulmonary torsion after nonpulmonary thoracotomy is extremely rare. We present a case of left upper pulmonary lobe torsion after resuscitative thoracotomy.

## CASE REPORT

**Figure 1:**
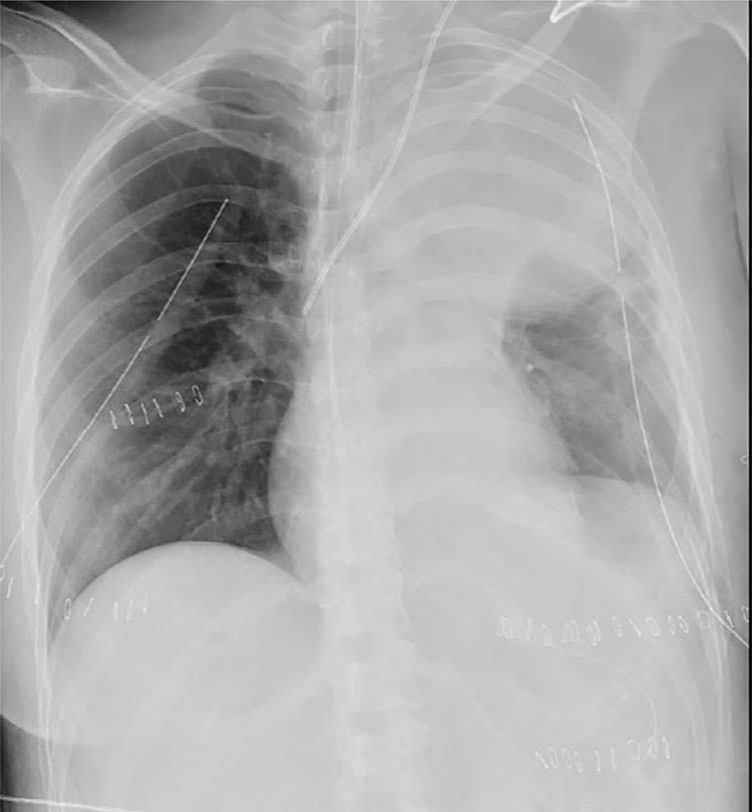
Chest roentgenogram of 2 days after initial emergency operation.

**Figure 2:**
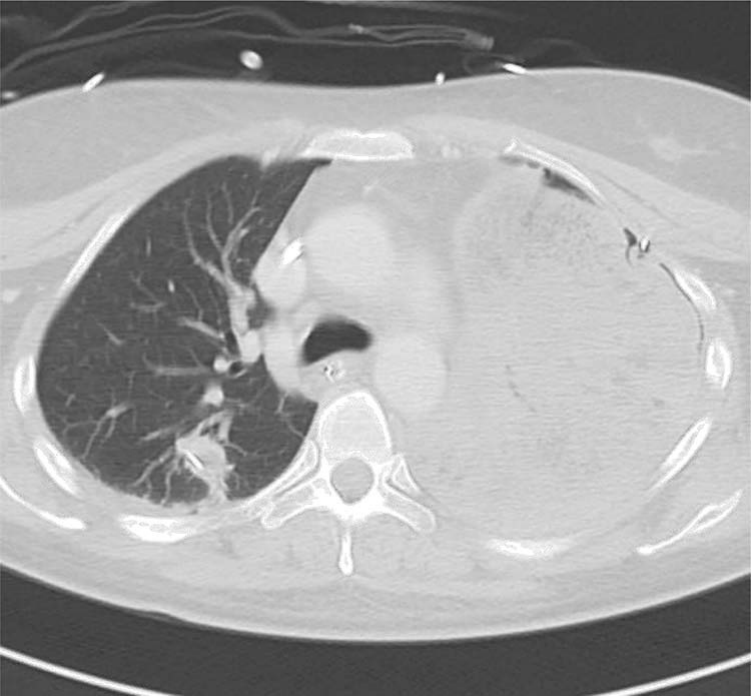
Computed tomography images of 2 days after initial emergency operation.

**Figure 3:**
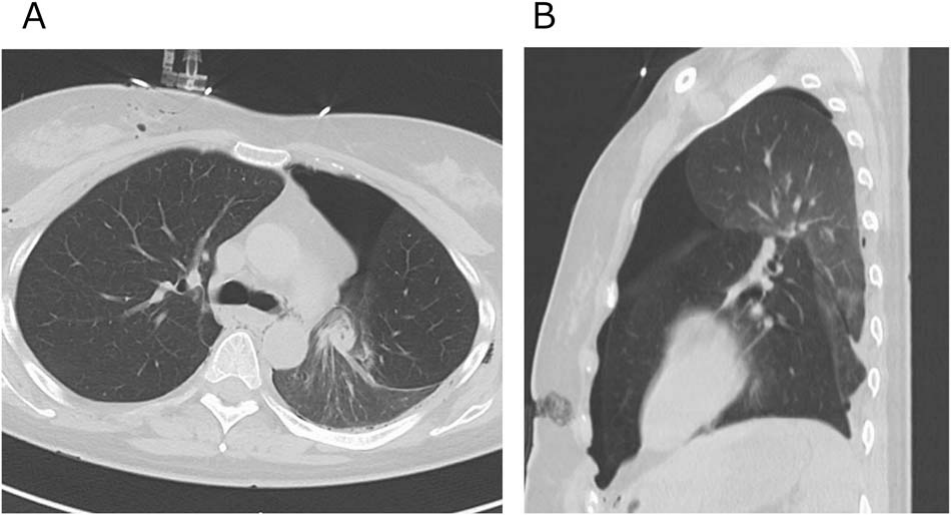
Axial (**A**) and sagittal (**B**) computed tomography images few hours after initial emergency operation.

A 38-year-old woman with schizophrenia committed suicide by a self-infliction of sharp force into the chest and abdomen. She presented to our hospital in a critical condition. On arrival in the emergency room (ER), she developed hemorrhagic shock, which leads to an emergent abdominal damage control surgery under general anesthesia. She lost cardiac activity in the operating room during exploratory laparotomy. A left-sided basal thoracostomy, cross-clamping of the descending thoracic aorta, and open cardiac massage were also performed. She received gauze packing at the bleeding point and temporarily close the abdomen and chest wall. After the surgery, she was transferred to the intensive care unit with mechanical ventilation and recovered from hemorrhagic shock. Two days later, a second-look laparotomy was performed. ER doctors verified the hemostasis and closed the patient’s abdomen and chest. On the same day, a chest radiograph revealed pulmonary atelectasis of the left upper lobe (Fig. 1). Computed tomography (CT) revealed alveolar bleeding, pneumonia and atelectasis of the left upper lobe (Fig. 2). Despite antibiotic therapy and repeated endobronchial sputum aspiration, radiologic findings were not improved. On the seventh postoperative day, she was referred to our department for suspected lung abscess. When compared to the chest CT images on the day of initial surgery, pulmonary torsion was suggested (Fig. 3A). The sagittal CT images were reconstructed, and the torsion of the left upper lung lobe was diagnosed (Fig. 3B). Therefore, an emergency left upper lobectomy was performed through a left thoracotomy. Intraoperatively, the left upper lung lobe was rotated clockwise and was hemorrhagically and congestive infarcted. Due to severe pulmonary congestion, it was difficult to obtain the surgical field of view. The pericardial sac was opened and the intrapericardial isolation of the left upper pulmonary vein was performed. To prevent separation of the thrombus and necrotic material in the left upper lobe, we first dissected the left upper pulmonary vein before resolving the torsion and performing the lobectomy. She had a complete pulmonary fissure. Moreover, only one first pulmonary artery branch had ruled the approximately pulmonary arterial flow of her left upper lobe. The left upper lobectomy was performed. The patient made an uneventful recovery.

## DISCUSSION

Pulmonary torsion is a rare but serious condition, most often observed after pulmonary resection, trauma and pneumothorax [1–3]. The incidence of pulmonary torsion after pulmonary resection has been reported as 0.089%–0.3% [3–6]. The pulmonary torsion after nonpulmonary thoracotomy is extremely rare [2, 7].

Felson *et al.* reported risk factors for pulmonary torsion; including an airless space, long free lobar pedicle, complete interlobar fissures resulting in loss of parenchymal bridge between contiguous lobes, pneumothorax and pleural effusion, heavy compact lung (due to atelectasis, consolidation or tumors) and transaction of the pulmonary ligaments [1]. Although the present case did not have other risk factors mentioned above except for a complete interlobar fissure, there is a possibility that she had other risk factors.

Both CT and bronchoscopy are the modalities of choice for diagnosis. Apostolakis and Hennink *et al.* stated that the most suggestive bronchoscopic findings included bronchial occlusion and fish mouth orifice [5, 6]. CT findings of pulmonary torsion were reported to be decreased aeration of torsion lung lobes, bronchial stenosis, or obstruction and pulmonary arteriovenous flexion [1, 6]. Although the present case received bronchoscopic sputum aspiration and CT scan, the diagnosis of pulmonary torsion was delayed. Pulmonary torsion might be easily misdiagnosed because of nonspecific clinical features, confusing imaging findings and the rarity of the condition [1]. Therefore, it is important to suspect the pulmonary torsion.

Only a limited number of cases with pulmonary torsion after nonpulmonary thoracotomy have been reported previously. Dai *et al.* reviewed previous publications of pulmonary torsions from January 1950 to December 2014 using three databases (PubMed, EMBASE and Web of Science) [7]. Shiomi *et al.* also reviewed the literature of pulmonary torsions [2]. Except for pulmonary resection, thoracic trauma and lung transplantation, there are only 19 cases of pulmonary torsion after nonpulmonary thoracotomy (esophageal cancer in seven, other esophageal diseases in six, congenital heart diseases in two, aortic dissection in two and mediastinal tumor in two patients) [2, 7]. To our knowledge, this is the first report of pulmonary torsion after resuscitative thoracotomy. Since the resuscitative thoracotomy was generally performed through lower intercostal space, it might be difficult to observe the pulmonary hilum and upper lobe. Therefore, surgeons should be aware of such rare complications after resuscitative thoracotomy.

When pulmonary torsion develops, it leads to rapid necrosis of the lung parenchyma [8]. Thus, pulmonary torsion needs to be diagnosed and managed promptly. Emergent surgery may be mandatory to save the patient.

## CONCLUSION

We experienced a rare case of pulmonary torsion of the left upper lobe after resuscitative thoracotomy.
